# Analysis of Host Immunological Response of Adenovirus-Based COVID-19 Vaccines

**DOI:** 10.3390/vaccines9080861

**Published:** 2021-08-04

**Authors:** Suzan Farhang-Sardroodi, Chapin S. Korosec, Samaneh Gholami, Morgan Craig, Iain R. Moyles, Mohammad Sajjad Ghaemi, Hsu Kiang Ooi, Jane M. Heffernan

**Affiliations:** 1 Modelling Infection and Immunity Lab, Mathematics Statistics, York University, Toronto, ON M3J 1P3, Canada; chapinskorosec@gmail.com (C.S.K.); s.gh3495@gmail.com (S.G.); 2 Centre for Disease Modelling (CDM), Mathematics Statistics, York University, Toronto, ON M3J 1P3, Canada; imoyles@yorku.ca; 3 Sainte-Justine University Hospital Research Centre and Department of Mathematics and Statistics, Université de Montréal, Montreal, QC H3T 1J4, Canada; morgan.craig@umontreal.ca; 4 Digital Technologies Research Centre, National Research Council Canada, Toronto, ON C1A 4P3, Canada; mghaemi@fields.utoronto.ca (M.S.G.); hsukiang.ooi@nrc-cnrc.gc.ca (H.K.O.)

**Keywords:** adenovirus-based vaccine, SARS-CoV-2, COVID-19, adaptive immune response, neutralizing antibody (NAb), IgG antibody, mathematical modeling

## Abstract

During the SARS-CoV-2 global pandemic, several vaccines, including mRNA and adenovirus vector approaches, have received emergency or full approval. However, supply chain logistics have hampered global vaccine delivery, which is impacting mass vaccination strategies. Recent studies have identified different strategies for vaccine dose administration so that supply constraints issues are diminished. These include increasing the time between consecutive doses in a two-dose vaccine regimen and reducing the dosage of the second dose. We consider both of these strategies in a mathematical modeling study of a non-replicating viral vector adenovirus vaccine in this work. We investigate the impact of different prime-boost strategies by quantifying their effects on immunological outcomes based on simple system of ordinary differential equations. The boost dose is administered either at a standard dose (SD) of 1000 or at a low dose (LD) of 500 or 250 vaccine particles. Results show dose-dependent immune response activity. Our model predictions show that by stretching the prime-boost interval to 18 or 20, in an SD/SD or SD/LD regimen, the minimum promoted antibody (Nab) response will be comparable with the neutralizing antibody level measured in COVID-19 recovered patients. Results also show that the minimum stimulated antibody in SD/SD regimen is identical with the high level observed in clinical trial data. We conclude that an SD/LD regimen may provide protective capacity, which will allow for conservation of vaccine doses.

## 1. Introduction

The spread of coronavirus disease 2019 (COVID-19), caused by severe acute respiratory syndrome coronavirus-2 (SARS-CoV-2), can be mitigated through safe and effective vaccines. Different vaccine types are currently being used to protect individuals from SARS-CoV-2 infection and disease. The four main types of COVID-19 vaccine in clinical trial includes whole virus, protein subunit, viral vector, and nucleic acid (RNA and DNA).

Limitations in vaccine supply, however, can affect the outcomes of the global vaccination campaign. Reductions in dose size, and a second dose delay whereby the second dose is delivered in a time frame beyond the manufacturer’s recommended schedule, can thus be considered, so that vaccine supply issues are diminished. In this work, we mathematically model adenovirus-based vaccines using a system of simple ordinary differential equations. The goals of our mathematical modeling study are two-fold, to (1) identify biological and vaccine characteristics that may allow for heightened and longer-lasting immune responses from vaccination, and (2) study the outcomes of a delayed second dose with the same or smaller dose size. The goals of this study are directly related to vaccine supply as we can determine if delaying and administering smaller second doses can provide immunological protection of similar magnitude to the recommended vaccine schedule (i.e., two similar-sized doses separated by 28 days).

The ODE-based model introduced in this work is based on the biological signaling pathway of the immune response to vaccination. The model includes both cellular and humoral immune system components, including vaccine particles, T helper cells, interferon-gamma (IFNγ), interleukin 6 (IL6), plasma B-cells, antibody, and cytotoxic T-cells. Model parameters were fit to clinical trial data for the COVID-19 ChAdOx1-S (AZD1222) vaccine developed by the University of Oxford and AstraZeneca [1].

Our results show evidence for a dose-dependent behavior of the immune system in response to an adenovirus-based vaccine [2]. Our results suggest that limiting the booster decreased antibody and cytotoxic T-cell levels as compared to regimens that follow the manufacturer’s recommended dose size and dosing schedule. Model results also show that, compared to clinical data [3], the model-predicted antibody level is comparable to the level attained by a patient with mild symptoms who recovers from COVID-19. Model predictions of attenuated IFNγ level by second shot delay and reduced dose size can provide a measure of vaccine efficacy and safety of a vaccine program with a delayed second dose.

This paper is organized as follows: In Section 2, we describe the mathematical model of the adaptive immune response. We then fit the model to available clinical trial data for the Oxford/AstraZenca vaccine [1]. A sensitivity analysis is then performed to determine model parameters that most affect peak values of the immune system outcomes from vaccination and, thus, the longevity of components of vaccine-induced immunity. Finally, we study the effects of delays in the second dose of the vaccine and the use of smaller-sized second doses. We conclude the paper in Section 4.

## 2. Model

**Figure 1:**
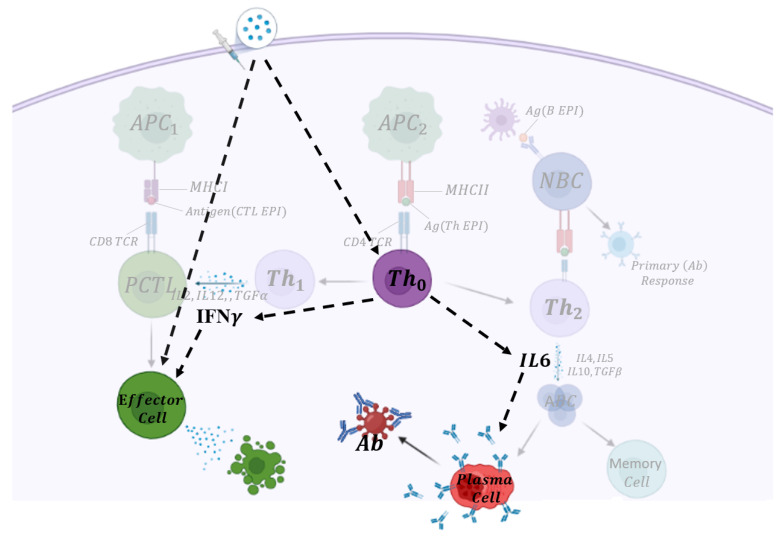
Vaccine-induced immune activation pathway for an adenovirus vaccine. Faint background: The subsequent downstream of signaling pathways activated through adaptive immunity when SARS-CoV-2 enters the human cell. Highlighted compartments describe vector-based vaccine-induced immune system stimulation that is modeled explicitly in this study. The dashed arrows show implicit communications between cells and cytokines, and the only solid arrow indicates the production of antibodies by B-cells.

The adaptive immune system is activated after exposure to an antigen either through vaccination or infection by a pathogen if the innate immune response is insufficient to stop the disease. Cell-mediated immunity, contributed by T-cells, and humoral immunity, controlled by activated B-cells, are components of the adaptive immune response that activate the immune system to protect the human body. These immune response components also generate memory T- and B-cells to protect an individual from future infection or disease. We have developed a mathematical model of an adenovirus vaccine that considers humoral and cell-mediated immune response mechanisms. The mechanisms of the cell-mediated immune response are illustrated in Figure 1, using bold and shaded components. Upon vaccination, vaccine particles will be recognized by components of the innate immune response, antigen-presenting cells (APCs, denoted here by APC1 and APC2, which are related to major histocompatibility complex (MHC) class 1 and 2 molecules, respectively). T helper type 0 cells (Th${}_{0}$) are activated through (APC2) and differentiate into Th${}_{1}$ and Th${}_{2}$ cells (central part). Cytotoxic T-cells (CTL, also called CD8 T-cells) can then be stimulated through the cytokine production from Th${}_{1}$ cells, including interleukins (IL2, IL12), transforming growth factor-alpha TGFα and Interferon IFNγ. Activated CTL differentiates into effector cells, which can then become memory CD8 T-cells. Th${}_{2}$ cells recognize the Th epitopes that are presented by B-cells through the MHC class II receptors. After being activated, Th${}_{2}$ cells secrete IL4, IL5, IL6, IL10, and TGFβ to stimulate B-cell activation and differentiation into plasma cells and memory B-cells (right part). The plasma cell produces neutralizing (NAb) antibody responsible for clearing the infection [4,5,6].

A simple network that reflects the entire diagram in Figure 1 is shown in bold. This simple network provides the basis of the mathematical model used in this study and has been chosen so to reduce the dimension of the system, given available parameters from the literature and the availability of the vaccine data. We explicitly consider T helper type 0 cells, plasma B-cells, antibody, cytotoxic T-cells, and two central cytokines, including IFNγ and IL6. An external stimulus, the vaccine, activates the immune response. The model consists of the following system of seven nonlinear ordinary differential equations.

(1a) $$\begin{array}{cc} \frac{dV}{dt} & =-\alpha_{16}VA-\gamma_{v}V \end{array}$$

(1b) $$\begin{array}{cc} \frac{dT}{dt} & =\mu_{21}V-\gamma_{t}T \end{array}$$

(1c) $$\begin{array}{cc} \frac{dF}{dt} & =\mu_{32}T-\gamma_{f}F-\alpha_{37}F\;C \end{array}$$

(1d) $$\begin{array}{cc} \frac{dI}{dt} & =\mu_{42}T-\gamma_{i}I-\alpha_{45}I\;B \end{array}$$

(1e) $$\begin{array}{cc} \frac{dB}{dt} & =\mu_{52}T+\alpha_{54}\left(\frac{I}{S_{i}+I}\right)B-\gamma_{b}B \end{array}$$

(1f) $$\begin{array}{cc} \frac{dA}{dt} & =\mu_{65}B-\gamma_{a}A-\alpha_{61}A\;V \end{array}$$

(1g) $$\begin{array}{cc} \frac{dC}{dt} & =\mu_{71}V+\alpha_{73}\left(\frac{F}{S_{f}+F}\right)C-\gamma_{c}C \end{array}$$

with variables summarized in Table 1, and 21 model parameters, listed in Table 2. Accordingly, parameters referring to variable production processes are denoted by $\mu_{ij}$, where *i* and *j* $=\;\left\{1,2,\cdots ,7\right\}$, corresponding to populations $\left\{V,T,F,I,B,A,C\right\}$, and denote the stimulated and stimulating populations, respectively. Parameters referring to the interaction of entities *i* and *j* are denoted by $\alpha_{ij}$. We note that $\alpha_{i,j}$ is not necessarily equal to $\alpha_{ji}$. Finally, parameters referring to the natural death of the population under consideration are defined by $\gamma_{k}$, where *k* in $\left\{v,t,f,i,b,a,c\right\}$, corresponding to the model variables.

In Equation (1a), vaccine particles are injected into the host with a predefined dosage. Their inhibition is described by natural decay and neutralization by antibody [7,8].

T helper cells are essential cells in that they are involved in activating the humoral and cell-mediated immune responses. T helper cells are activated by specialized antigen-presenting cells (APCs) through the primary histocompatibility class II/peptide complexes. Model (1) considers T helper type 0 cells only (we do not consider differentiated T-cell dynamics). See Equation (1b). We also simplify the model by ignoring the explicit population of APCs and instead assume that the Th${}_{0}$ population activation is proportional to the vaccine particles in the system (i.e., we assume that the APC population is proportional to the vaccine particles count).

IFNγ is a type-II IFN that plays a crucial role in regulating the adaptive immune response. It is produced by a wide variety of lymphocytes, including CD4+, CD8+, and regulatory T (Treg) cells, B-cells, and NK cells. Although numerous cells can express IFNγ, it is mainly secreted by T-cells, and it is the defining cytokine of Th${}_{1}$ helper cells [9,10]. Accordingly, and based on the signaling pathway introduced by Figure 1 here, in Equation (1c), we define the source term of IFNγ ($\mu_{32}T$) to be the Th${}_{0}$ cells in the system. We also assume that IFNγ can be degraded or removed in the system by (1) Th${}_{0}$ cell surface binding (for mitotic and stimulation signals in cytotoxic T-cell, *C*, proliferation [11]), and (2) natural decay.

We also explicity model IL6, another cytokine in the system (Equation (1d)). IL6 is a pleiotropic cytokine [12,13] produced by many different T-cell types, including T- and B-cells [14], that has a pivotal role in the activation and stimulation of the immune response [15]. IL6 also plays an important role in antibody production [16]. IL6 has a wide range of functions and acts as a B-cell stimulatory factor to induce antibody production [17]. Elevated IL6 levels are found in COVID-19 patients with mild and severe symptoms [18,19,20,21,22,23,24,25,26,27,28,29,30,31] implying that IL6, alongside other cytokines, can be of prognostic value in these patients [32]. In our mathematical model, IL6 is considered to be secreted indirectly by Th${}_{0}$ cells and is partially absorbed for stimulation signals in B-cell priming.

Plasma B-cells are long-lived, non-proliferating cells arising from B-cell differentiation, stimulated by interaction with T helper cells. Activated plasma B-cells produce neutralizing antibody, which are responsible for clearing the infection. In Equation (1e) we consider an indirect activation of plasma B-cells by Th${}_{0}$ cells at rate $\mu_{52}T$, and by IL6, which is assumed to have an adjuvanted role in stimulation, $\alpha_{54}\left(\frac{I}{S_{i}+I}\right)B$, where $\alpha_{54}$ is a recruitment rate and $S_{i}$ is a saturation constant. Plasma B-cells die naturally at rate $\gamma_{b}$.

Humoral immunity is an antibody-mediated response that occurs when plasma B-cells are activated. In Equation (1f) we assume that antibody production is proportional to the number of plasma B-cells, by rate $\mu_{65}$. We also assume that antibodies are degraded at a rate $\gamma_{a}$, or removed through vaccine binding at rate $\alpha_{61}$. They can be lost to the system through vaccine particle binding ($\alpha_{61}A\;V$). Note that the simulated antibody, without specialization, is entirely the neutralizing antibody (Nabs), which is responsible for defending cells from pathogens or infectious particles by neutralizing its biological effects.

Finally, like the activation of Th${}_{0}$ cells, in Equation (1g), we assume that cytotoxic T-cells (also known as cytotoxic T-lymphocytes, CTL, and activated CD8 T-cells) priming is proportional to the number of vaccine cell particles ($\mu_{71}V$). We also assume that IFNγ can stimulate further cytotoxic effector T-cells (term $\alpha_{73}\left(\frac{F}{S_{f}+F}\right)C$) [9,33]. Finally, cytotoxic T-cells die at rate $\gamma_{c}$.

The initial conditions for all activated cells and cytokines are zero T(0)=F(0)=I(0)=B(0)=A(0)=C(0)=0, assuming that we are starting in a system with no activated immune response. The first and second doses are entered into the system using an initial condition of V(0)=1000, and an impulse of 1000, 500 or 250 vaccine particles is provided to the system at the time of the second dose. The standard dose size used here is chosen to be 1000 vaccine particles. This is an arbitrary value. When a smaller or larger dose size is chosen, parameters $\mu_{21}$, $\alpha_{61}$ and $\mu_{71}$ are simply rescaled.

### 2.1. Parameter Fitting

The vaccine on which we base our parameters is the one produced by AstraZeneca/ Oxford [1], an adenovirus-based SARS-CoV-2 vaccine that has been approved in many countries, such as the United Kingdom, Bangladesh, Egypt, Australia, Canada, Thailand, Malaysia, Philippines, South Korea and so on. We parameterize the model by fixing some parameter values from the literature and others to the vaccine trial data using a grid search method. Some parameters had limited data availability, and their values are chosen (see Table 2). Sensitivity analyses are performed to assess variations in model outcomes due to changes in the fixed and chosen parameters. The final fit of the model to the vaccine trial data was defined by the minimum of the root-mean-squared error (RMSE), which considers differences between the IFNγ and antibody data for SARS-CoV-2 IgG response, and the model-predicted values for these values. Clinical trial data for one and two doses of the vaccine are both considered.

We note that the parameters of the modeled neutralizing antibody response are fit to IgG clinical data. Although IgG is the most common antibody (70–75% of all human immunoglobulins found in the plasma [34,35]), we must consider a proportionality between IgG and the antibody population. We thus assumed that IgG =ε NAbs, and the antibody equation in Model (1) becomes

$$\frac{dIg}{dt}=\mu_{65}\varepsilon B-\gamma_{a}Ig-\alpha_{61}Ig\;V\;.$$

Consequently, we consider $\mu_{65}\varepsilon$ as one parameter in the model fit to the vaccine trial data.

### 2.2. Sensitivity Analysis

We employ sensitivity analysis methods that include Latin hypercube sampling (LHS) [36,37] and partial rank correlation coefficients (PRCC) to study the effects of parameter variation on key model outcomes of interest. We use 10,000 samples from a uniform distribution over parameter ranges within the interval with median = parameter value, listed in Table 2, minimum = 0.5*parameter and maximum = 1.5*parameter. PRCC values are calculated on model outcomes associated with a strong vaccine-induced immune response. We choose to study the peak magnitude of each model variable, as peak value correlates with a longer-lasting immune response. We note that PRCC values with a magnitude close to unity indicate that the parameter has the highest attainable significant impact on the model output [38]. A value greater than 0.5 is assumed to be significant [38]. Additionally, PRCC values can be negatively (negative sign) or positively correlated with a model outcome [39].

## 3. Results

**Figure 2:**
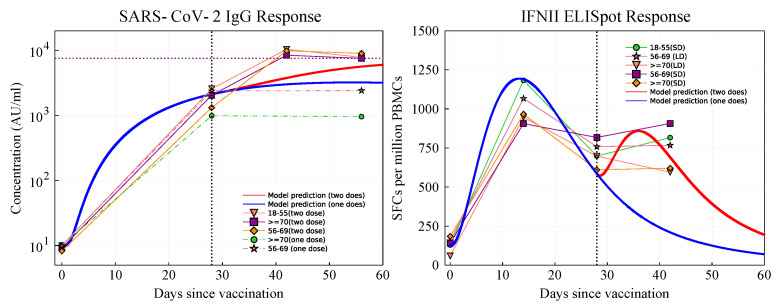
Antibody and IFNγ fit to the clinical trial data [1]. Blue and red solid lines: predicted results for participants who received one (blue) or two doses (red), with a boost dose at day 28 (shown by black vertical dashed lines). Left: The purple horizontal dashed line shows the maximum stimulated antibody level post-boost.

The model fit to the IgG and IFNγ data [1] is shown in Figure 2. We observe that post priming, the model predicted antibody level is comparable to the clinical trial data for the 56–69 (one dose) age group, and it lies in the upper range of the antibody levels reported by [1]. After boosting (red line), the antibody level increases to about $10^{4}$ titer, which lies close to the lower end of the clinical trial measurements.

The model-predicted result of IFNγ after the prime is also consistent with clinical data and boosted by the second dose injection. The resulting IFNγ level predicted by the model lies close to the lower values measured in the clinical trial at days 28 and 42.

### 3.1. Sensitivity Analysis

**Figure 3:**
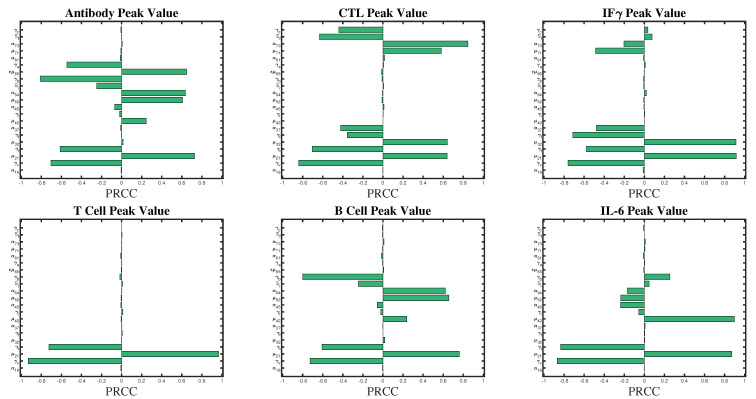
Sensitivity analysis of Model (1) using 10,000 iterations of a Latin hypercube sampling (LHS) method with a partial rank correlation coefficient (PRCC). PRCC values with magnitude close to unity indicate that the parameter has a strong impact on the model output [38].

A sensitivity analysis is performed to assess changes in model outcomes as parameter values are varied. We examine the sensitivity of the peak values of T helper cells, IFNγ, interleukin, B-cells, antibody, and cytotoxic T-cells. We are interested in determining what parameters maximize peak values so that longer-lasting immune outcomes can be realized from vaccination (assuming that longer-lasting immunity correlates with increased peak value). Results of the LHS/PRCC analysis are shown in Figure 3 for all model parameters. Significant parameters affecting peak values, with an absolute value of PRCC > 0.5, are listed in Table 3. A monotonic relationship between the outcomes and the parameter values is confirmed in all cases.

Generally, we find that increases in peak value correlate with increases in stimulation and secretion, and decrease with increases in death and decay rates. Peak antibody and plasma B-cells both have a high sensitivity to $\gamma_{b}$, the B-cell natural death rate, $\alpha_{54}$, the B-cell stimulation rate by IL−6 and $\mu_{52}$, the B-cell activation rate by Th${}_{0}$. Peak CTL is very sensitive to $\alpha_{73}$, simulation rate by IFNγ,$\mu_{32}$, IFNγ stimulation rate by Th${}_{0}$, $S_{f}$, CTL duplication threshold due to IFNγ, which is a chosen parameter, and activation by virus particles, $\mu_{71}$. Variations in secretion rates $\mu_{32}$ and $\mu_{42}$ significantly affect the peak values of interferon and interleukin, respectively.

We note that variation in $\mu_{21}$, $\gamma_{t}$ and $\gamma_{v}$ significantly affects all populations. This is an intuitive result as $\mu_{21}$, $\gamma_{t}$, and $\gamma_{v}$ all affect the peak Th${}_{0}$ value, and the Th${}_{0}$ population stimulates the rest of the immune response.

We note that $\mu_{21}$ is is a chosen parameter. Although variation in its value significantly affects all population peak values, since it is related to the activation rate of the Th${}_{0}$ population, which activates the rest of the immune response, it is always countered by sensitivity to $\gamma_{v}$ and $\gamma_{t}$, which are parameters informed by the literature. Given a constant activation and proliferation capacity of Th${}_{0}$ cells, an increase in $\mu_{21}$ would require an increase in $\gamma_{t}$ of similar magnitude. Therefore, we conclude that sensitivity to this parameter is limited.

Lastly, we note that the only other chosen parameter value that significantly affects any model outcome is $S_{f}$, the saturation constant of the CTL population. The parameter ranks 6th in significance related to peak CTL value only, suggesting that sensitivity to this parameter is not a concern.

### 3.2. Mechanism of Vaccine-Induced Immunity with Booster Delay and Sparing

**Figure 4:**
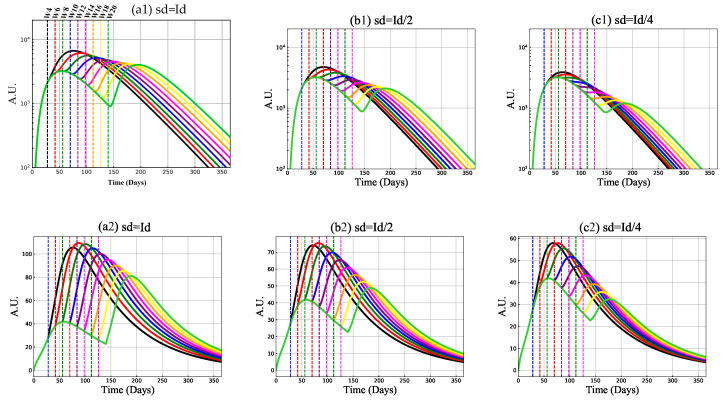
Antibody (IgG) and CTL outcomes with standard (SD) and low dose (LD), with and without delay. Model predictions of antibody, first row, and cytotoxic T-cells, second row, with second dose vaccination on days: 28 (week:4), 42 (week:6), 56 (week:8), 70 (week:10), 84 (week:12), 98 (week:14), 112 (week:16), 126 (week:18), and 140 (week:20, shown by colored vertical dashed lines.). The second dose value (sd) in panel (**a**) is the same as the initial dose (id) value: (sd = Id = 1000 vaccine particles), in panel (**b**) it is decreased by half (sd = Id/2 = 500 vaccine particles), and in panel (**c**) is decreased by a quarter (sd = Id/4 = 250 particles).

We now apply Model (1) in a study of reduced second dose volume, and its delay. We consider several different scenarios based on varying assumptions on boosting (second dose). We first provide the system with a standard dose (SD) of $10^{3}$ vaccine particles. We then provide a SD second dose, a low dose (LD) of 500 vaccine particles, or a LD of 250 vaccine particles. The second dose is injected into the system 28, 42, 56, 70, 84, 98, 112, 126, or 140 days later (corresponding to 4, 6, 8, 10, 12, 14, 16, 18, and 20 weeks between doses). Figure 4 shows the antibody and CTL populations generated from the model considering all of these cases.

#### 3.2.1. Antibody and Cytotoxic T-Cell Responses

Model predictions suggest quantitative differences in neutralizing antibody and cytotoxic T-cell responses stimulated by the vaccine administration by an SD or LD boost 4 to 20 weeks post priming, see Figure 4. By comparing the SD/SD and SD/LD regimens, we find that higher peaks of antibodies and CTL are achieved for the second dose when the SD dose is provided—viewing the second peak in each panel of Figure 4, the maximum activated antibody level decreases from panel (a) to panels (b) and (c), the maximum stimulated CTL reduces from 80–100 in the SD/SD regimen (panel (a)) to 50–70 (panel (b)) and 30–60 (panel (c)) in SD/LD regimens. We, however, also observe that a higher antibody or CTL peak after the second dose, compared to the first dose peak, may not be achievable if the doses are too far apart (long delays in second dose), or the dose is too small. With respect to antibodies, the peak after the second dose is always greater than the first dose peak if an SD is used. When an LD is used, the time between doses must be shorter. For the CTL, a higher second peak can be achieved under all scenarios using an SD and an LD = SD/2, but the peak after the second dose may not surpass the first peak if an LD = SD/4 is used (shorter times between doses are needed).

Considering the CTL population we can also observe that shorter times between doses do not necessarily result in larger CTL peak values. Here, we observe that a time-frame of 4 weeks between doses is not optimal. Generally, we find that the second-dose induced antibody enhancement is increased if the prime-boost time interval is short. The differing outcomes between the antibody and CTL populations may be explained by the fact that CTL activity might be required to account for lower levels of antibodies that cannot neutralize the virus particle efficiently.

#### 3.2.2. Cytokines, B and Th${}_{0}$ Cell Responses

**Figure 5:**
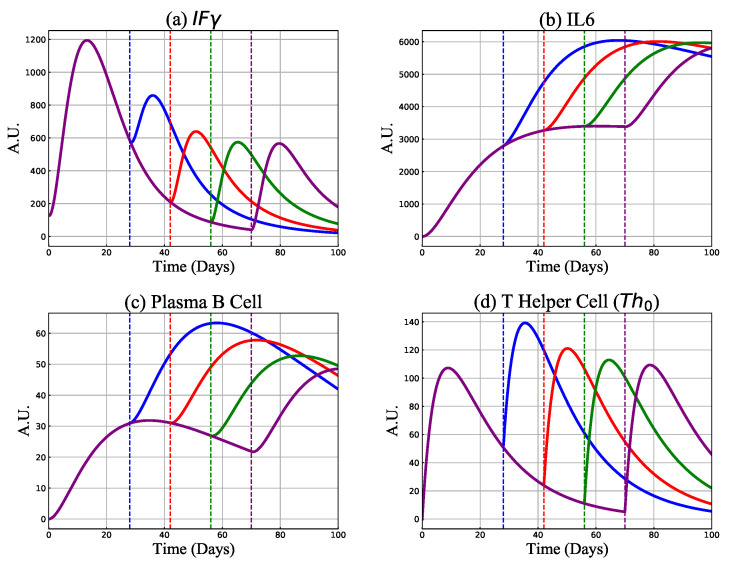
Model predictions of IFNγ, IL-6, plasma B-cells and T helper type 0 cells Th${}_{0}$ for the received boost dose (1000 vaccine particles) on days 28 (blue curve), 42 (red curve), 56 (green curve) and 70 (purple curve). The vertical dashed lines show the second dose injection days.

Here we investigate the model predictions of proinflammatory cytokines, including IFNγ and IL6, alongside plasma B- and Th${}_{0}$-cells for four different prime-boost intervals (containing 4, 6, 8, and 10 weeks) in SD/SD regimen. Results are shown in Figure 5. We find that prolonging the time interval between doses reduces levels of IFNγ but increases IL6 levels. We also observe that the plasma B-cell count increases, but that a Th${}_{0}$ enhancement can only be achieved if the time between doses is less than 70 days.

#### 3.2.3. Protective Capacity

An important question that we must consider is whether the model-predicted antibody and CTL levels would protect against SARS-CoV-2 infection by existing or new emerging variants. Our models results cannot comment on direct protection, but we can compare the model outcomes to antibody levels in recovered COVID-19 individuals. In [3], the authors measured SARS-Cov-2-specific neutralizing antibody in plasma from 175 recovered COVID-19 patients with mild symptoms. They reported that 39% of the patients have medium-high antibody 1000-25000 titers (ID50), that 30% have less than 500 antibody titers, and 14% have titers greater than 2500. Figure 4 shows that after the second dose injection for the SD/SD regimen the maximum stimulated IgG antibody for short prime-boost intervals, such as 4, 6, 8, 10 weeks, is much higher than a 2500 titer (≈$10^{4}$). The maximum stimulated antibody, however, decreases as the time between doses increases. At 20 weeks between doses, the antibody level reaches ≈4000 upon boosting. Interestingly, for the regimen SD/LD, with the second dose value at SD/2, the promoted antibody level up to an 18-week delay is greater than 2500 and is about the same level at 20 weeks between doses (≈2500). For the last scenario where the second dose is a quarter of the prime dose, the minimum activated antibody level is ≈1000 for a long time-frame between doses (week 18, or 20).

We must now consider neutralizing antibodies to through the parameter varepsilon. From [35], if we assume ε=0.7, we find that the minimum promoted neutralizing antibody through different second-dose injection weeks (from week = 4, ...., 20) for (i) SD/SD regimen is ≈5714, (ii) SD/LD regimen with LD = SD/2 is approximated by 3571, and (iii) SD/LD with LD = SD/4 is ≈1429. Considering a worst-case scenario with ε=0.3 for IgG percentage, we have ≈13,333, 8333 and 3333 minimum antibody titer levels of stimulated neutralizing antibody. We thus predict that using even the pessimistic range of neutralizing antibody achieves the same level of high and medium-to-high neutralizing antibodies as observed in COVID-19 recovered individuals.

## 4. Discussion

In this work, we have employed a mathematical model to study the vaccine-induced adaptive immune response through cell-mediated and humoral (antibody-mediated) immunity given an adenovirus vaccine [1]. Using a set of nonlinear ordinary differential equations, we present a new model of vaccine-induced immunity that is parameterized with the clinical trial data for the COVID-19 ChAdOx1-S (AZD1222) vaccine. The model parameters are determined by grid search over parameter ranges to minimize the RMSE of IFNγ and antibody functions. In addition to the fitted parameters, the model includes some chosen parameters. Our sensitivity analysis in Figure 3 shows that variations in these parameters do not significantly affect model peak values of the model parameters, except $mu_{21}$, the activation rate of Th${}_{0}$ by the vaccine particles.

Our model predictions for IFNγ and antibody are consistent with the clinical trial data of the adenovirus-based Oxford vaccine [1]. Recent studies have explored scenarios for reduced vaccine dose size, to consider if vaccine supply is scarce. In [40] the authors studied the effects of reducing the prime dose of a SARS-CoV-2 adenovirus-based vaccine in a mouse model. Their in vivo experiments demonstrated that mice initially primed with a low-dose (LD) vaccine significantly exhibited a higher level of the immune response. In another study, Geoffroy et al. looked at the effects of increasing the time interval between doses, using an SIR epidemiological model [41]. We have considered SD/LD cases with varying time frames between doses. Our results demonstrate that an enhanced immune response can be realized in some immune response populations, whereas the antibody response is best if doses are given 28 days (4 weeks) apart.

Our mathematical model does not take into account memory B-cells and T-cells, instead focusing on correlations between such memory cells and the model peak plasma B- and Th${}_{0}$-cells. With more clinical data availability, future extension may include stimulation of memory cells. The inclusion of memory cells is a course for future work.

We have analyzed different scenarios given a second dose of vaccine that is delayed or reduced in size. Our model predictions show that either limiting the second dose or increasing the prime-boost time interval leads to an attenuated adaptive immune response. However, in agreement with previous clinical findings [3], the model-predicted antibody achieves levels lie in the same range as the neutralizing antibody in 39% of COVID-19 recovered patients. Hence, a delayed second dose in combination with smaller doses sizes may allow for sufficient dose allocation to meet specific population vaccination targets while maintaining vaccine efficacy. We note that we do not consider specific vaccine efficacy here, and simply compare neutralizing antibody levels, which are a measure of protection. A study of vaccine efficacy against different strains of SARS-CoV-2 is a course for future work.

It is important to note that [1] observed similar outcomes in LD/LD scenarios in their clinical trial compared to SD/SD individuals, across different age groups, given a 28-day interval between doses. We have not considered this case here, but we plan to in future work.

## Figures and Tables

**Table 1:** Model variables.

Variable	Definition
*V*	Vaccine cell
*T*	T helper type 0 cell (Th${}_{0}$)
*F*	Interferon gamma (IFNγ)
*I*	Interleukin 6 (IL−6)
*B*	Plasma B-cell
*A*	Antibody
*C*	Cytotoxic T-cell

**Table 2:** Model Parameters.

Parameter	Definition	Value	Unit	Comment
$\alpha_{16}$	Vaccine neutralizing rate by antibody molecules	1 $\times \;10^{-6}$	day${}^{-1}$(a.u.)${}^{-1}$	Handle et al., 2018
$\gamma_{v}$	Vaccine clearance rate	0.2	day${}^{-1}$	Cao et al., 2016
$\mu_{21}$	Th${}_{0}$ cells activation rate by vaccine particles	0.035	day${}^{-1}$	Chosen
$\gamma_{t}$	Th${}_{0}$ cells natural death rate	0.055	day${}^{-1}$	Cao et al., 2016
$\mu_{32}$	IFNγ stimulation rate by Th${}_{0}$	2.55	day${}^{-1}$	Fitted
$\gamma_{f}$	IFNγ natural degradation rate	0.13	day${}^{-1}$	Fitted
$\alpha_{37}$	IFNγ absorption rate by CTL for mitotic signals	0.006	day${}^{-1}$(a.u.)${}^{-1}$	Fitted
$\mu_{42}$	IL6 release rate by Th${}_{0}$	1.3	day${}^{-1}$	Fitted
$\gamma_{i}$	IL6 natural degradation rate	0.0008	day${}^{-1}$	Chosen
$\alpha_{45}$	IL6 absorption rate by B-cells for mitotic signals	0.0001	day${}^{-1}$(a.u.)${}^{-1}$	Fitted
$\mu_{52}$	B-cell activation rate by Th${}_{0}$	0.02	day${}^{-1}$	Fitted
$\alpha_{54}$	B-cell stimulation rate by IL	0.05	day${}^{-1}$(a.u.)${}^{-1}$	Fitted
$S_{i}$	B-cell duplication threshold due to IL	1000	a.u.	Chosen
$\gamma_{b}$	B-cell natural death rate	0.06	day${}^{-1}$	Fitted
$\varepsilon \mu_{65}$	Released Ab rate by B-cells	7	day${}^{-1}$	Fitted
$\gamma_{a}$	Ab natural degradation rate	0.06	day${}^{-1}$	Fitted
$\alpha_{61}$	Ab - V cells binding rate	1 $\times \;10^{-7}$	day${}^{-1}$(a.u.)${}^{-1}$	Chosen
$\mu_{71}$	CTL activation rate by vaccine	0.002	day${}^{-1}$	Fitted
$\alpha_{73}$	CTL stimulation rate by IFNγ	0.09	day${}^{-1}$(a.u.)${}^{-1}$	Fitted
$S_{f}$	CTL duplication threshold due to IFNγ	600	a.u.	Chosen
$\gamma_{c}$	CTL natural death rate	0.01	day${}^{-1}$	Wang et al., 2016

**Table 3:** Parameter sensitivity with absolute value of PRCC≥0.5.

Variable	Parameter	Absolute PRCC Value
A (Antibody)	$\gamma_{b}$	0.8<PRCC<0.9
	$\mu_{21}$	≈0.8
	$\gamma_{v}$	≈0.8
	$\varepsilon \mu_{65}$	≈0.7
	$\alpha_{54}$	0.6<PRCC<0.7
	$\gamma_{t}$	≈0.6
	$\mu_{52}$	≈0.6
	$\gamma_{a}$	0.5≤PRCC<0.6
	$\alpha_{73}$	0.8<PRCC<0.9
	$\gamma_{v}$	0.8<PRCC<0.9
	$\gamma_{t}$	≈0.7
C (CTL)	$\mu_{21}$	0.6<PRCC<0.7
	$\mu_{32}$	0.6<PRCC<0.7
	$S_{f}$	≈0.7
	$\mu_{71}$	≈0.6
F (IFNγ)	$\mu_{21}$	≈0.9
	$\mu_{32}$	≈0.9
	$\gamma_{v}$	0.7<PRCC<0.8
	$\gamma_{t}$	≈0.6
	$\mu_{71}$	≈0.5
	$\alpha_{37}$	≈0.5
	$\mu_{21}$	≈1
T (Th${}_{0}$)	$\gamma_{v}$	≈1
	$\gamma_{t}$	≈0.7
Plasma B	$\mu_{21}$	≈0.8
	$\gamma_{b}$	≈0.8
	$\gamma_{v}$	0.7<PRCC<0.8
	$\mu_{52}$	≈0.7
	$\alpha_{54}$	0.6<PRCC<0.7
	$\gamma_{t}$	≈0.6
I (IL6)	$\mu_{42}$	≈0.9
	$\mu_{21}$	≈0.9
	$\gamma_{v}$	0.8<PRCC<0.9
	$\gamma_{t}$	0.8<PRCC<0.9

## Data Availability

Not applicable.

## Abbreviations

The following abbreviations are used in this manuscript:

APC	Antigen-Presenting Cell
MHC	Major Histocompatibility Complex
$Th_{0}$	T Helper Cell Type 0
$Th_{1}$	T Helper Cell Type 1
$Th_{2}$	T Helper Cell Type 2
NK	Natural Killer Cell
CTL	Cytotoxic T Lymphocyte
IL	Interleukin
IFN	Interferon
TGF	Transforming Growth Factor
NAb	Neutralizing Antibody
IgG	Immunoglobulin G
